# Diabetes and Functional Impairment in Osteoarthritis: Investigating Correlations, Associations, and Progression

**DOI:** 10.7759/cureus.64493

**Published:** 2024-07-13

**Authors:** Surya Kalamegam, Pankaj B Shah, Akshaya Damodaran

**Affiliations:** 1 Community Medicine, Panimalar Medical College Hospital & Research Institute, Chennai, IND; 2 Community Medicine, Sri Ramachandra Institute of Higher Education and Research, Chennai, IND; 3 General Medicine, Sri Ramachandra Institute of Higher Education and Research, Chennai, IND

**Keywords:** knee joint, degeneration, functional impairment, diabetes, osteoarthritis

## Abstract

Introduction

Diabetes and osteoarthritis (OA) are prevalent chronic conditions, often occurring concurrently and complicating patient management. While the individual impact of each condition on functional impairment is well documented, their combined effect remains poorly understood. This study aims to elucidate the relationship between diabetes and OA-related functional impairment.

Methodology

This was a cross-sectional study of 290 participants with unilateral knee OA. Their demographic, clinical, and diabetes data were collected. Functional impairment was assessed using the Western Ontario and McMaster Universities Osteoarthritis Index-Center for Rheumatic Diseases (WOMAC-CRD). Statistical analyses investigated the relationships between diabetes, OA severity, and functional impairment.

Result

Diabetic participants showed significantly worse physical function and overall disability, with lower WOMAC-CRD scores. Mean WOMAC-CRD pain scores were 6.46 (SD = 1.088) and 6.48 (SD = 1.101) for the diabetic and non-diabetic groups, respectively. Mean WOMAC-CRD stiffness scores were 6.48 (SD = 1.101) and 6.56 (SD = 1.083) for diabetic and non-diabetic groups. Diabetic participants had a mean WOMAC-CRD physical function score of 55.93 (SD = 2.484), compared to 64.02 (SD = 2.542) for non-diabetic participants. The mean total WOMAC score was 68.80 (SD = 2.857) for diabetic participants and 77.06 (SD = 2.933) for non-diabetic participants. Longer diabetes duration correlated negatively with physical function and total WOMAC scores.

Discussion

The findings suggest that diabetes exacerbates functional impairment in OA patients, particularly affecting physical function and overall disability. Chronic inflammation and the accumulation of advanced glycation end-products may contribute to the observed deterioration in joint function.

Conclusion

Integrated management strategies addressing both diabetes and OA are essential for optimizing patient care.

## Introduction

Osteoarthritis (OA), the most common form of arthritis, is characterized by the progressive degradation of articular cartilage and changes in the subchondral bone and synovium, leading to pain, stiffness, and loss of joint function [1]. It predominantly affects the elderly, with the knee and hip joints being the most commonly involved [2]. Conversely, diabetes mellitus (DM), particularly type 2 diabetes (T2D), is a long-term metabolic illness characterized by hyperglycemia brought on by insulin resistance or insufficient insulin secretion. It affects many body systems and raises the risk of several complications, such as nephropathy, neuropathy, and cardiovascular diseases [3].

The coexistence of OA and T2D is increasingly observed in clinical practice, raising questions about potential interactions and mutual influences between these conditions [4]. Epidemiological research has repeatedly demonstrated that people with T2D have a greater prevalence of OA than people without the condition. This link points to a potential two-way interaction in which osteoarthritis may be influenced by diabetes in terms of its incidence, severity, and development, and vice versa [5]. However, the exact nature of this relationship, including the underlying mechanisms and clinical implications, remains an area of active investigation.

Several shared risk factors contribute to the overlapping prevalence of OA and T2D. Obesity is a primary common factor, as it not only increases the mechanical load on weight-bearing joints, exacerbating OA but also contributes to the development and progression of insulin resistance [6]. Additionally, both conditions are more prevalent with advancing age and are associated with a sedentary lifestyle. However, beyond these shared risk factors, specific pathophysiological mechanisms may link diabetes to the progression of OA.

Chronic inflammation is a pivotal component in the pathogenesis of both OA and T2D. In diabetes, persistent hyperglycemia leads to “a state of chronic low-grade inflammation, characterized by elevated levels of pro-inflammatory cytokines such as tumor necrosis factor-alpha (TNF-α), interleukin-1 beta (IL-1β), and IL-6. These cytokines can accelerate the degradation of cartilage and synovial tissue in joints”, thus exacerbating OA [7]. Furthermore, advanced glycation end-products (AGEs), which accumulate in hyperglycemic conditions, can modify the structural properties of cartilage, making it stiffer and more susceptible to damage. Advanced glycation end-products also activate specific receptors (receptors for advanced glycation end-products (RAGE)) that promote inflammatory pathways, contributing to the pathophysiology of OA [8].

The functional impairment associated with OA, such as pain, reduced mobility, and decreased quality of life, is often more pronounced in individuals with diabetes [9]. Diabetic neuropathy, a common complication of diabetes, can alter pain perception and proprioception, leading to abnormal joint loading and an increased risk of joint damage [10]. Additionally, vascular complications associated with diabetes, such as peripheral arterial disease, can impair blood flow to joint tissues, compromising their ability to heal and repair [11]. Insulin resistance and poor glycemic control can further reduce muscle strength and endurance, crucial for maintaining joint stability and function [12].

The progression of OA in the context of diabetes is often accelerated, with diabetic patients experiencing more rapid cartilage loss and joint damage [13]. Hyperglycemia-induced oxidative stress and the resultant inflammatory response contribute to this accelerated progression [14].

Creating efficient treatment plans requires an understanding of the complex link between diabetes and OA. The purpose of this study is to further investigate the relationships, development, and correlations between diabetes and functional impairment in individuals with OA.

## Materials and methods

After receiving clearance from the institutional ethics committee of Sri Ramachandra Institute of Higher Education and Research, Chennai, India, a cross-sectional study was carried out (approval number: CSP-MED/22/JAN/73/15). Based on the American College of Rheumatology's (ACR) Clinical Classification Criteria for Osteoarthritis of the Knee, 290 individuals in all had a diagnosis of unilateral OA of the knee. Written informed consent was acquired from consenting subjects after they were fully informed about the nature and goal of the study.

Individuals 45 years of age and older who had been diagnosed with unilateral knee OA based on ACR criteria were included in the study. Exclusions from the research included those with other forms of arthritis, major comorbidities that limited movement, or cognitive impairments that interfered with evaluations.

Demographic information was recorded for all participants. To measure functional impairment in individuals with knee OA, the Western Ontario and McMaster Universities Osteoarthritis Index-Center for Rheumatic Diseases (WOMAC-CRD) score, an Indian adaptation of the WOMAC [15], was employed. The WOMAC-CRD score comprises three components: pain, stiffness, and difficulty out of a total of 24 questions related to knee and hip functioning. On a scale of 0-4, the exam questions are rated as follows: none (0), mild (1), moderate (2), severe (3), and extreme (4). Each subscale's scores are added together, and the potential ranges are 0-20 for pain, 0-8 for stiffness, and 0-68 for physical function. The validity and reliability of the WOMAC-CRD score have been acknowledged. Near-current data on the patients' glycated hemoglobin (HbA1c) levels, fasting blood glucose, and the duration of their diabetes were used to evaluate their glycemic control in T2D cases.

IBM SPSS Statistics software for Windows, version 20.0 (IBM Corp., Armonk, NY) was used to analyze the data. Descriptive statistics for every variable that was measured were computed. The link between variables was evaluated using the Pearson correlation test; in particular, the relationship between diabetes-related parameters and the WOMAC-CRD index's measures of functional impairment and OA severity was examined. A probability threshold of 5% was deemed statistically significant.

Before each person was included in the study, their informed consent was obtained. The study's participants were guaranteed the privacy of their information and the freedom to leave at any stage without compromising their access to medical treatment.

## Results

The study included 290 participants diagnosed with unilateral OA of the knee, distributed across various age groups. The age distribution is displayed in Table 1. Of the participants, 34 (11.7%) were between the ages of 55 and 60 years, 70 (24.1%) were between the ages of 61 and 65 years, 63 (21.7%) were between the ages of 66 and 70 years, 72 (24.8%) were between the ages of 71 and 75 years, and 51 (17.6%) were between the ages of 76 and 80 years.

**Table 1:** Sociodemographic distribution of the study participants

Variable	Category	Frequency	Percentage	Cumulative percentage
Age	55 - 60 years	34	11.70%	11.70%
	61 - 65 years	70	24.10%	35.90%
	66 - 70 years	63	21.70%	57.60%
	71 - 75 years	72	24.80%	82.40%
	76 - 80 years	51	17.60%	100.00%
	Total	290	100.00%	
BMI	Underweight (<18.5 kg/m^2^ )	29	10.00%	10.00%
	Normal weight (18.5 - 24.5kg/m^2^ )	28	9.70%	19.70%
	Overweight (25 - 29.5kg/m^2^ )	63	21.70%	41.40%
	Obesity Class 1 (30 - 34.5 kg/m^2^)	71	24.50%	65.90%
	Obesity Class 2 (35 - 39.5 kg/m^2^)	52	17.90%	83.80%
	Obesity Class 3 (>40 kg/m^2^)	47	16.20%	100.00%
	Total	290	100.00%	
Sex	Male	156	53.80%	53.80%
	Female	134	46.20%	100.00%
	Total	290	100.00%	
Socioeconomic status (BG Prasad Classification)	Upper class	41	14.10%	14.10%
	Upper middle class	98	33.80%	47.90%
	Middle class	85	29.30%	77.20%
	Lower middle class	52	17.90%	95.20%
	Lower class	14	4.80%	100.00%
	Total	290	100.00%	
Diabetes status	Diabetes present	157	54.10%	54.10%
	Diabetes absent	133	45.90%	45.90%
	Total	290	100.00%	100.00%

There was a small male predominance in the gender distribution, with 156 men (53.8%) and 134 females (46.2%). In terms of socioeconomic status, the participants were distributed as follows: 41 participants (14.1%) were in the upper class, 98 participants (33.8%) in the upper middle class, 85 participants (29.3%) in the middle class, 52 participants (17.9%) in the lower middle class, and 14 participants (4.8%) in the lower class.

The individuals' body mass indices (BMIs) differed greatly. Ten percent of the participants (10.0%) were classified as underweight (BMI <18.5 kg/m^2^), 9% had normal weight (BMI 18.5-24.5 kg/m^2^), 67% had an overweight BMI 25-29.5 kg/m^2^, 27% were classified as overweight (BMI 25-29.5 kg/m^2^), 21% were classified as obese class 1 (BMI 30-34.5 kg/m^2^), 17% were classified as obese class 2 (BMI 35-39.5 kg/m^2^), and 16% were classified as obese class 3 (BMI >40 kg/m2). In terms of diabetes status, 133 people (45.9%) did not have the disease, whereas 157 people (54.1%) did.

The following was found by looking at the descriptive statistics for the WOMAC-CRD scores in Table 2: The average physical function score was 59.64 (SD = 4.754), the average WOMAC-CRD pain score was 6.46 (SD = 1.088), the average stiffness score was 6.48 (SD = 1.101), and the average total WOMAC score was 72.59 (SD = 5.03495). Table 3 showed no statistically significant variations in the stiffness and pain levels between the subjects with and without diabetes. For those with diabetes, the mean WOMAC-CRD pain score was 6.45 (SD = 1.123), and for those without diabetes, it was 6.48 (SD = 1.049) (p =.783). For those with diabetes, the mean stiffness score was 6.42 (SD = 1.116), and for those without diabetes, it was 6.56 (SD = 1.083, p =.295). On the other hand, the overall WOMAC scores and physical function showed significant variations. Participants with diabetes had a mean physical function score of 55.93 (SD = 2.484), compared to 64.02 (SD = 2.542) for those without diabetes (p <.001). Participants with diabetes had a total WOMAC score of 68.80 (SD = 2.857), whereas those without diabetes had a score of 77.06 (SD = 2.933, p <.001).

**Table 2:** Descriptive statistics of the WOMAC-CRD score WOMAC-CRD: Western Ontario and McMaster Universities Osteoarthritis Index-Center for Rheumatic Diseases

Variable	N	Minimum	Maximum	Mean	Std. Deviation
WOMAC-CRD pain	290	5	8	6.46	1.088
WOMAC-CRD stiffness	290	5	8	6.48	1.101
WOMAC-CRD physical function score	290	52	68	59.64	4.754
WOMAC-CRD total score	290	63.00	84.00	72.5862	5.03495
Valid N (listwise)	290				

**Table 3:** Independent t-test association of diabetes with functional disability among osteoarthritis patients WOMAC-CRD: Western Ontario and McMaster Universities Osteoarthritis Index-Center for Rheumatic Diseases

Measure	Diabetes status	N	Mean	Std. Deviation	Mean difference	95% CI lower	95% CI upper	P-value
WOMAC-CRD pain	Diabetes present	157	6.45	1.123	-0.035	-0.288	0.217	0.783
WOMAC-CRD pain	Diabetes absent	133	6.48	1.049	-0.035	-0.287	0.216	0.782
WOMAC-CRD stiffness	Diabetes present	157	6.42	1.116	-0.136	-0.391	0.119	0.295
WOMAC-CRD stiffness	Diabetes absent	133	6.56	1.083	-0.136	-0.391	0.119	0.294
WOMAC-CRD physical function score	Diabetes present	157	55.93	2.484	-8.093	-8.675	-7.51	0
WOMAC-CRD physical function score	Diabetes absent	133	64.02	2.542	-8.093	-8.676	-7.509	0
WOMAC total score	Diabetes present	157	68.7962	2.85715	-8.26397	-8.93482	-7.59313	0
WOMAC total score	Diabetes absent	133	77.0602	2.93299	-8.26397	-8.93638	-7.59156	0

**Figure 1:**
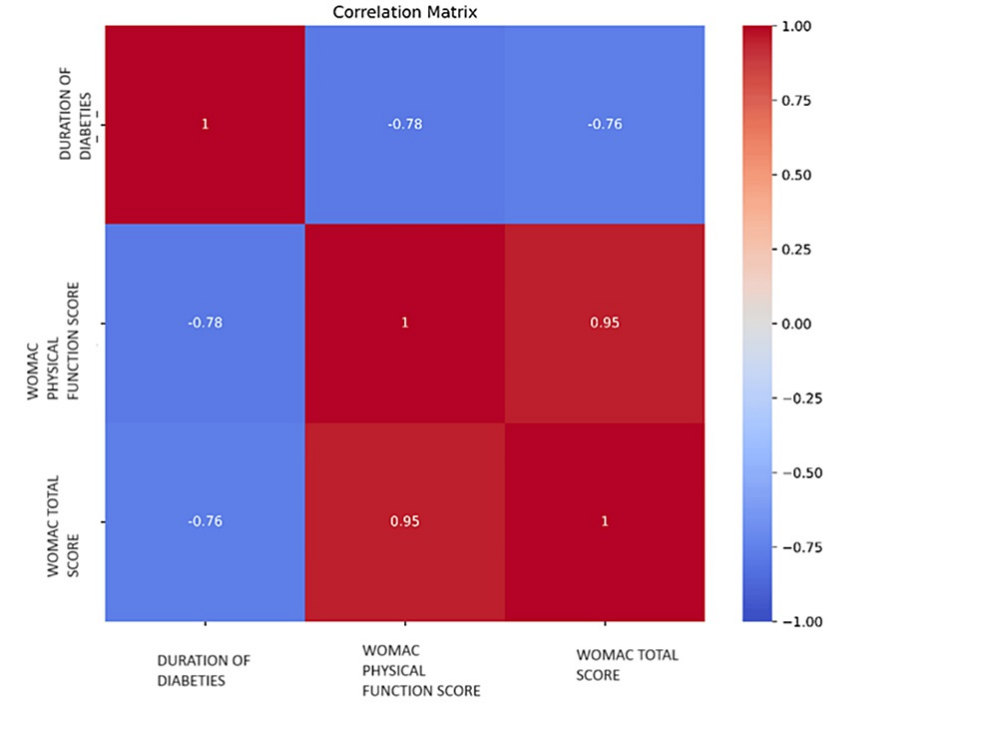
A heatmap illustrates the correlations between the duration of diabetes, the WOMAC-CRD physical function score, and the WOMAC-CRD total score. WOMAC-CRD: Western Ontario and McMaster Universities Osteoarthritis Index-Center for Rheumatic Diseases

Figure 1 presents a heatmap of the correlation analysis results, which indicates a statistically significant negative relationship between the length of diabetes and the physical function score (r = -.782, p <.001) as well as the overall WOMAC score (r = -.762, p <.001). Furthermore, there was a significant positive association (r =.950, p <.001) found between the physical function score and the overall WOMAC score. The color bar in the graph in the heatmap represents the correlation coefficients between the variables. The color gradient typically ranges from blue to red, where blue indicates a positive correlation and red indicates a negative correlation. In this specific heatmap, the color bar shows that the correlation between the duration of diabetes and the WOMAC-CRD physical function score is strongly negative (deep red). The correlation between the duration of diabetes and the WOMAC-CRD total score is also strongly negative (deep red). The correlation between the WOMAC-CRD physical function score and the WOMAC-CRD total score is strongly positive (deep blue). This color bar helps in visually interpreting the strength and direction of the correlations between the variables.

**Figure 2:**
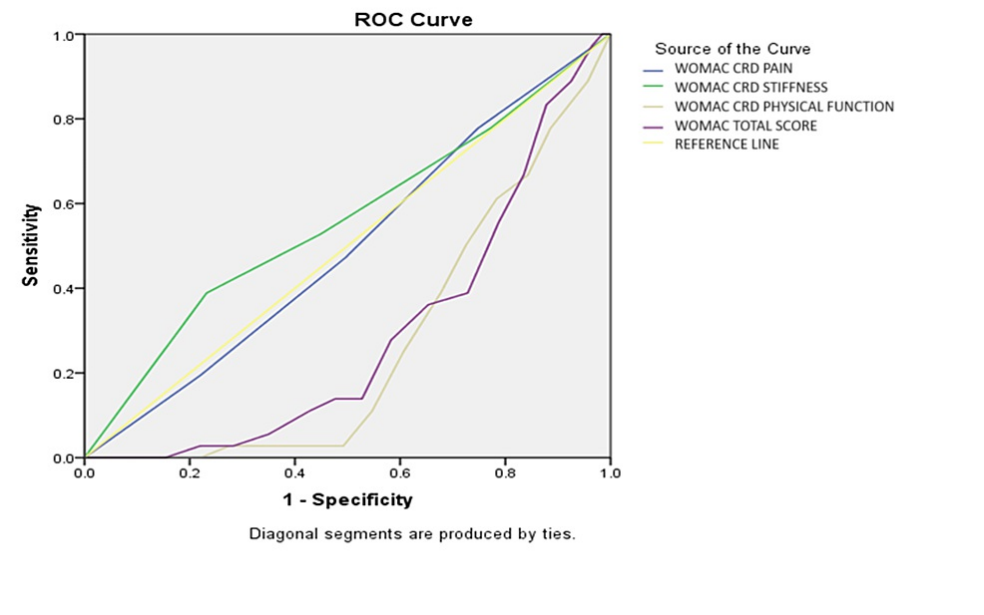
Receiver operating characteristic (ROC) analysis was conducted to assess the discriminative ability of various test result variables in predicting the duration of diabetes among osteoarthritis patients. WOMAC-CRD: Western Ontario and McMaster Universities Osteoarthritis Index-Center for Rheumatic Diseases

The test result variables (WOMAC-CRD pain, stiffness, physical function score, and total WOMAC score) varied in their ability to discriminate between participants who had had diabetes for less than seven years and those who had it for longer, according to receiver operating characteristic (ROC) curve analysis in Figure 2. The WOMAC-CRD pain (.495), WOMAC-CRD stiffness (.558), WOMAC-CRD physical function score (.267), and overall WOMAC score (.289) were the area under the curve (AUC) values. These findings imply that while the pain and stiffness ratings are less predictive of diabetes duration, the WOMAC-CRD physical function score and overall WOMAC score are.

In summary, the study found that diabetes significantly impacts physical function and overall disability in individuals with knee OA, with longer diabetes duration correlating with greater functional impairment.

## Discussion

The current study set out to look at the relationships, development, and correlations of functional impairment in individuals with and without OA and diabetes. The study's conclusions, which show notable variations in physical function and total handicap, offer insightful information on the intricate interactions between diabetes and OA.

The demographic distribution of the study participants revealed a balanced representation across different age groups, with a slight male predominance (53.8%). The age distribution was relatively even, though a substantial proportion of participants were aged between 61 and 75 years, consistent with the typical age range for OA onset. The BMI data indicated a high prevalence of overweight and obesity among the participants, aligning with known risk factors for both OA and diabetes.

The results demonstrated a clear association between diabetes and increased functional impairment in OA patients. Although the pain and stiffness scores were not significantly different between participants with and without diabetes, there were substantial differences in the physical function and total WOMAC-CRD scores. Participants with diabetes had significantly worse physical function (mean score of 55.93) compared to those without diabetes (mean score of 64.02) and a lower total WOMAC-CRD score (68.80 vs. 77.06, respectively). These findings suggest that, while diabetes may not exacerbate the subjective experience of pain and stiffness in OA, it does have a profound impact on physical function and overall disability.

The negative correlation between the duration of diabetes and both the physical function score (r = -.782) and the total WOMAC score (r = -.762) underscores the progressive nature of diabetes-related complications in OA patients. A longer duration of diabetes was associated with greater functional impairment, indicating that chronic hyperglycemia and its systemic effects may contribute to the deterioration of joint function over time. This highlights the importance of early and effective diabetes management to potentially mitigate its impact on OA progression.

The role of chronic inflammation and the build-up of AGEs in diabetics is one reasonable explanation for the functional deficits that have been reported. Diabetes is frequently associated with elevated levels of inflammatory cytokines (e.g., TNF-α, IL-1β, and IL-6) and AGEs, which have been linked to the pathophysiology of both diabetes and OA. These biochemical mediators can worsen the symptoms of OA and contribute to the noted loss of physical function by causing cartilage degradation, synovial inflammation, and joint discomfort.

The study also considered socioeconomic status (SES) as a variable. Most of the participants belonged to the middle class and upper middle class groups. The results of this investigation have very significant therapeutic ramifications. They first emphasize the necessity of coordinated management plans that take diabetes and OA into account. Healthcare professionals should take into consideration integrating diabetes management into the overall treatment plan for patients with OA in light of the increased risk of functional impairment in this patient population. This might entail stricter glycemic management, lifestyle adjustments, and focused therapies to lessen inflammation and avoid joint injury.

Our study aligns with previous research, such as that of Dubey et al. (2018), who identified a significant association between DM and knee OA among both type 1 diabetes (T1D) and T2D patients. They found a strong association of knee OA with T1D (odds ratio (OR): 1.40) and T2D (OR: 2.75), underscoring the significant impact of diabetes on joint health. Interestingly, their findings revealed that the association between T2D and knee OA was more pronounced in non-obese individuals, contrasting with the common perception that obesity is a primary driver of OA in diabetic patients [16].

Eitner et al. (2021) further emphasized the detrimental effect of DM on osteoarthritic knee pain, independent of obesity and other confounders. Their study demonstrated that individuals with both OA and DM reported worse knee pain and overall physical and mental health status. The negative influence of DM on knee pain and health status persisted even after adjusting for age, sex, BMI, and radiographic severity, highlighting DM as an independent risk factor for heightened OA symptoms [17].

Neumann et al. (2019) provided additional evidence of accelerated knee joint structural degeneration in diabetic individuals over a four-year period. They observed significantly greater increases in cartilage and meniscus lesions among diabetics compared to non-diabetics, suggesting a more rapid deterioration of knee joint structure in the presence of diabetes. This accelerated degeneration underscores the need for targeted interventions to slow OA progression in diabetic patients [18]. 

Eymard et al. (2015) noted that T2D was a significant predictor of joint space narrowing (JSN) in men with established knee OA, indicating that DM contributes to the radiographic progression of OA. Their findings also suggested that other metabolic factors, including metabolic syndrome (MetS), did not significantly influence JSN, further isolating diabetes as a critical factor in OA progression [19].

Louati et al. (2015) conducted a meta-analysis that reinforced the association between DM and OA, reporting a high prevalence of OA among diabetic patients and vice versa. Their analysis suggested that DM is an independent risk factor for OA, even after adjusting for BMI, strengthening the argument that diabetes exacerbates OA beyond the influence of obesity alone [20]. 

Rios Arce et al. (2022) discussed the complex interplay between DM and OA, noting conflicting results in animal and human studies. They highlighted the need for future research to account for various confounding factors, such as type of diabetes, age, biological sex, and BMI. This comprehensive approach is crucial for developing more effective monitoring and treatment strategies for patients at risk of both DM and OA [21]. 

Our findings contribute to the growing body of evidence that diabetes significantly exacerbates functional impairment in OA patients, particularly affecting physical function and overall disability. The negative correlation between the duration of diabetes and physical function scores underscores the progressive nature of this impairment. This underscores the importance of early and effective diabetes management to mitigate its impact on OA progression and improve patient outcomes.

Limitations

This study's limitations include a cross-sectional design, reliance on self-reported data, and a lack of longitudinal follow-up. Additionally, the study did not account for confounders such as medication use and lifestyle.

## Conclusions

This study emphasizes how diabetes significantly affects the functional disability experienced by OA patients. The results underscore the necessity of comprehensive care strategies that tackle both ailments and stress the significance of prompt and efficient diabetes control to lessen its negative consequences on the advancement of OA. Understanding the complex interactions between diabetes and OA can help medical professionals create more thorough and efficient treatment programs that enhance patient outcomes and quality of life.
